# Link between the causative genes of holoprosencephaly: Zic2 directly regulates *Tgif1* expression

**DOI:** 10.1038/s41598-018-20242-2

**Published:** 2018-02-01

**Authors:** Akira Ishiguro, Minoru Hatayama, Maky I. Otsuka, Jun Aruga

**Affiliations:** 1 Laboratory for Behavioral and Developmental Disorders, RIKEN Brain Science Institute (BSI), Wako-shi, Saitama, 351-0198 Japan; 2 Research Center for Micro-Nano Technology, University of Hosei, Koganei, Tokyo, 184-0003 Japan; 3 Department of Medical Pharmacology, Nagasaki University Institute of Biomedical Sciences, Nagasaki, Nagasaki, 852-8523 Japan

## Abstract

One of the causal genes for holoprosencephaly (HPE) is *ZIC2* (HPE5). It belongs to the zinc finger protein of the cerebellum (Zic) family of genes that share a C2H2-type zinc finger domain, similar to the *GLI* family of genes. In order to clarify the role of Zic2 in gene regulation, we searched for its direct target genes using chromatin immunoprecipitation (ChIP). We identified *TGIF1* (HPE4), another holoprosencephaly-causative gene in humans. We identified Zic2-binding sites (ZBS) on the 5′ flanking region of *Tgif1* by *in vitro* DNA binding assays. ZBS were essential for Zic2-dependent transcriptional activation in reporter gene assays. Zic2 showed a higher affinity to ZBS than GLI-binding sequences. Zic2-binding to the cis-regulatory element near the *Tgif1* promoter may be involved in the mechanism underlying forebrain development and incidences of HPE.

## Introduction

Holoprosencephaly (HPE) is known as a common forebrain defect in human development^1,2^. At least 13 chromosomal loci are associated with nonsyndromic HPE^2,3^. *SHH*, *ZIC2*, *SIX3*, and *TGIF1* have been identified to be four causative genes in the HPE loci (*HPE3, HPE5, HPE2*, and *HPE4*, respectively) and have been investigated with respect to clinical spectrum^4^ and genetic interactions^5^. Among the major HPE-associated genes, the roles of *TGIF1* and *ZIC2* in forebrain development have been elusive until recently^1^. However, recent studies have revealed clues as to its roles in forebrain development.

*TGIF1* encodes a member of three-amino-acid loop extension (TALE) superfamily of homeodomain proteins. TGIF1 regulates downstream TGF-β signalling as a negative coregulator of Smad proteins^6–8^, and it also regulates retinoic acid (RA) signalling by binding to the cis-regulatory element of RA-controlling genes^9–11^. Subsequently, TGF-β and RA-signalling may be regulated in the context of TGIF1-mediated forebrain developmental control as indicated by knockdown of transforming growth-interacting factors in mouse^12^ and zebrafish models^13^, respectively. Furthermore, mice lacking *Tgif1* and the related *Tgif2* show HPE-like abnormalities with defects in the SHH signalling pathway partly independent of Nodal/TGF-β signalling^14,15^.

*ZIC2* belongs to the Zic family of zinc finger proteins that play various critical developmental roles^16,17^. Zic2 can act as a transcriptional regulator^18^ and helps in enhancer priming^19–21^. It can also form molecular complexes containing DNA-dependent protein kinase (DNA-PK) and RNA helicase A^22,23^, or chromatin remodelling complexes containing MBD3 and nucleosome remodelling deacetylase (NuRD)^20^. ZIC2 can also interact with GLI^24^ and SMAD proteins^25^, which helps in the modulation of SHH and Nodal TGF-β signalling, respectively. In mice, a *Zic2* knockdown mutation causes HPE-like brain abnormalities^26^, and a missense mutation in *Zic2* caused exencephaly with abnormal expression of organizer markers, impaired prechordal plate (PrCP) development^27^, and impaired Nodal signalling^25^. In zebrafish, a Zic2-related gene, *zic1*, controls midline formation and forebrain patterning by regulating Nodal, SHH, and RA signalling^28^. Thus, forebrain defects and affected signalling cascades could be caused by altered gene function of the overlapping roles of Zic2 and Tgif1.

In this study, we have shown that mouse Zic2 can directly bind to the 5′ flanking region of *Tgif1* to regulate *Tgif1* expression, and that a Zic2-Tgif1 linkage can be implicated in forebrain development and HPE.

## Results

### Isolation of direct target genes of Zic2

We performed ChIP to identify target genes of Zic2. We overeproduced N-terminally FLAG-Human influenza hemagglutinin-tagged Zic2 (F-HA-Zic2) in the A40 mouse cerebellar granule cell line^29^. F-HA-Zic2-target DNA fragment complexes were purified by sequential anti-FLAG and anti-HA immunoprecipitations. The co-precipitated DNA fragments were purified and cloned, and their sequences were determined. We identified 13 Zic2-binding sequences in the 5′ region of known genes (Supplementary Table S1). We also recently validated these results with ChIP-seq results obtained using mouse ES cells (GSE61188)^20^ or cerebellar granule neurons (GSE60731)^19^. It was observed that all of the identified targets were included in the ChIP-seq peaks (Supplementary Fig. S1).

### Zic2 protein recognizes a region upstream of the Tgif1 gene locus

**Figure 1:**
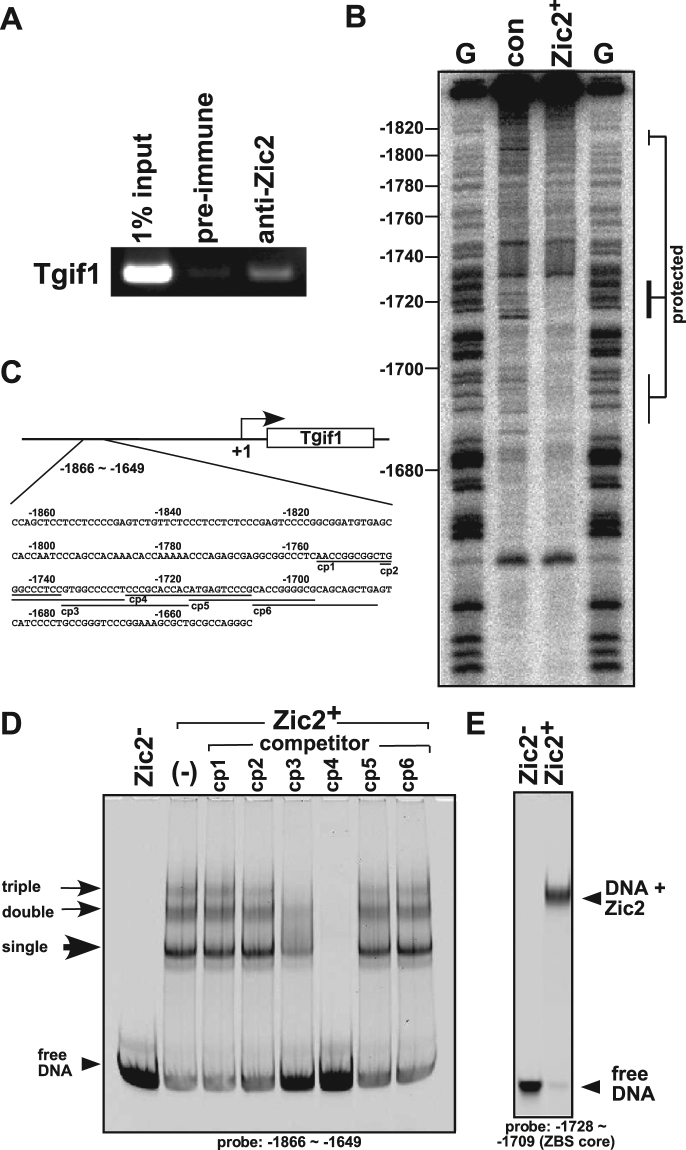
Zic2 binds to the *Tgif1* enhancer region. (**A**) Ethidium bromide-stained agarose gel showing the ChIP-PCR analysis. ChIP was performed with anti-Zic2 antiserum or preimmune rabbit serum. The same amount of cell extract was prepared without crosslinking and then purified, and 1% of total DNA mixture was used as a control. (**B**) DNA footprinting analysis, using DNase I, showing Zic2-binding sites as protected regions indicated at the right side. G indicates the sequence marker, which was digested at the dGTP end. Positions from the transcriptional start site are indicated on the left side. The reaction was performed in the presence or absence of Zic2 protein. (**C**) The sequence from –1866 to −1649 from the *Tgif1* transcriptional start site (+1). (**D**) EMS assay. To confirm the Zic2 binding sites, an IRD700 end labelled probe (−1866 to −1649) was incubated with Zic2 in the presence or absence of an unlabelled competitor (cp1–cp6). (**E**) EMS assay showing Zic2 binds to a restricted short fragment probe (−1728 to −1709; Zic2 binding site core).

We noticed that the Zic2-binding sequences included the 5′ flanking region of *Tgif1*, which is another HPE-causative gene. To address the possible linkage between the HPE-causative genes, we focused on Zic2 binding to the *Tgif1* 5′ flanking region. The original sequence bound by Zic2 harboured in the region of −1866 to −1649 from the transcriptional start site (Fig. 1). The binding of A40-endogenous Zic2 to this sequence was detected by a ChIP-PCR assay using anti-Zic2 antibody and PCR primers for this region (Fig. 1A). To identify all Zic2 binding sites, a DNA footprinting analysis using DNase I was performed within the cloned *Tgif1* 5′ flanking region. We found that one region (−1730 to −1710) was strongly protected, and two regions (−1830 to −1800 and −1700 to −1670) were weakly protected by the addition of Zic2 (Fig. 1B). The specific binding was then analysed by an electrophoretic gel mobility shift (EMS) assay using an IRD700 end-labelled duplex DNA probe containing the −1866 to −1649 region (Fig. 1C). Three bands were observed when the probe was incubated with the Zic2 (Fig. 1D). This result along with the result of the DNA footprinting assay (Fig. 1D, left side) indicate single, double, and triple Zic2 binding to the DNA probe. To detect the exact binding sites, we tried to disrupt the Zic2-DNA complexes using unlabeled short duplex DNA competitors (cp1–cp6). The overshifted bands were completely disappeared in the presence of the competitor cp4 (−1728 to −1709) and partially disappeared by cp3 (−1738 to −1719) (Fig. 1D). We also defined two additional weak binding sites using two different IRD700 labelled probes (−1709 to −1649 and −1866 to −1760) and several competitors (Supplementary Fig. S3). Both binding sites were confirmed by the competitor’s sensitivity at positions −1688 to −1669 and −1830 to −1811 (Supplementary Fig. S2). To evaluate the core binding affinity of ZIC2 at the region −1728 to −1709, we used an IRD700 end-labelled short probe. The majority of DNA probes were over shifted due to the addition of Zic2 (Fig. 1E). These results indicated that Zic2 binds to 3 sites in the −1866 to −1649 region (Zic2 binding sites, ZBS), with a core ZIC2 binding site between −1728 and −1709.

### ZBS are essential for Zic2 dependent transcriptional activation

**Figure 2:**
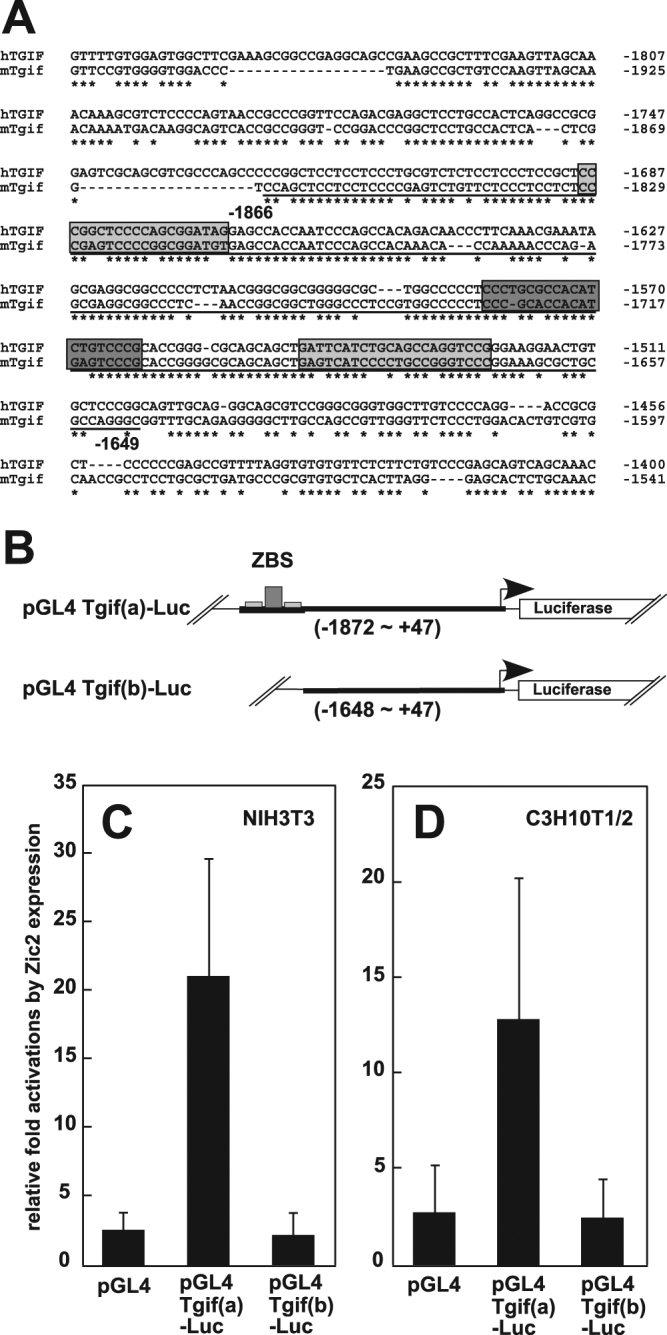
ZBS are essential for Zic2-dependent transcriptional activation. (**A**) Alignment between the human and mouse Zic2 binding-5′ flanking region of *Tgif1* gene. The underline indicates the originally cloned region via ChIP cloning. The dark gray box and two light gray boxes indicate Zic2 binding regions. Conserved nucleotides are shown by (*). (**B**) *Tgif1* enhancer – luciferase constructs. Although pGL4 Tgif(a) consists of the region from −1872 to +47 of *Tgif1*, pGL4 Tgif(b) lacks −1872 to −1649 containing the ZBS. (**C**,**D**) Zic2 overexpression enhances luciferase activity in a ZBS-dependent manner in NIH3T3 and C3H10T1/2 cell lines. All activities were normalized to Renilla luciferase activity. The averages and standard errors for three samples from three independent experiments are shown.

The regions containing ZBS in the *Tgif1* 5′ flanking region are well conserved between mice and humans (Fig. 2A), where a clear peak is observed for the ZIC2 binding site-containing region of the *TGIF1* promoter in ChIP-seq experiments using GFP-tagged human ZIC2 and human embryonic kidney 293 cells (GSE52523)^30^ (Supplementary Fig. S1).

To analyse Zic2-dependent regulation of *Tgif1* transcription, we constructed two luciferase reporter plasmids, pGL4 Tgif(a)-Luc and pGL4 Tgif(b)-Luc, that contain the *Tgif1* 5′ flanking region −1872 to +47 (containing ZBS) and −1648 to +47 (lacking ZBS) upstream of a luciferase gene (Fig. 2B), respectively. We compared the luciferase activity in cells transfected with the plasmids, in the presence or absence of Zic2 using NIH3T3 and C3H10T1/2 cell lines. As expected, the luciferase activity in NIH3T3 and C3H10T1/2 cells transfected with pGL4 Tgif(a)-Luc was 20- and 12-fold higher, respectively (Fig. 2C and D, respectively), by the coexpression of *Zic2*. In contrast, the luciferase activity in NIH3T3 and C3H10T1/2 cells transfected with pGL4 Tgif(b) remained unchanged (Fig. 2C and D, respectively), and had a similar luciferase expression level to that observed in cells transfected with the pGL4 control plasmid. These results strongly suggest that the ZBS are essential for Zic2-dependent transcriptional activation of *Tgif1* in these cells.

### ZBS are recognized by Zic2 but not by GLI

**Figure 3:**
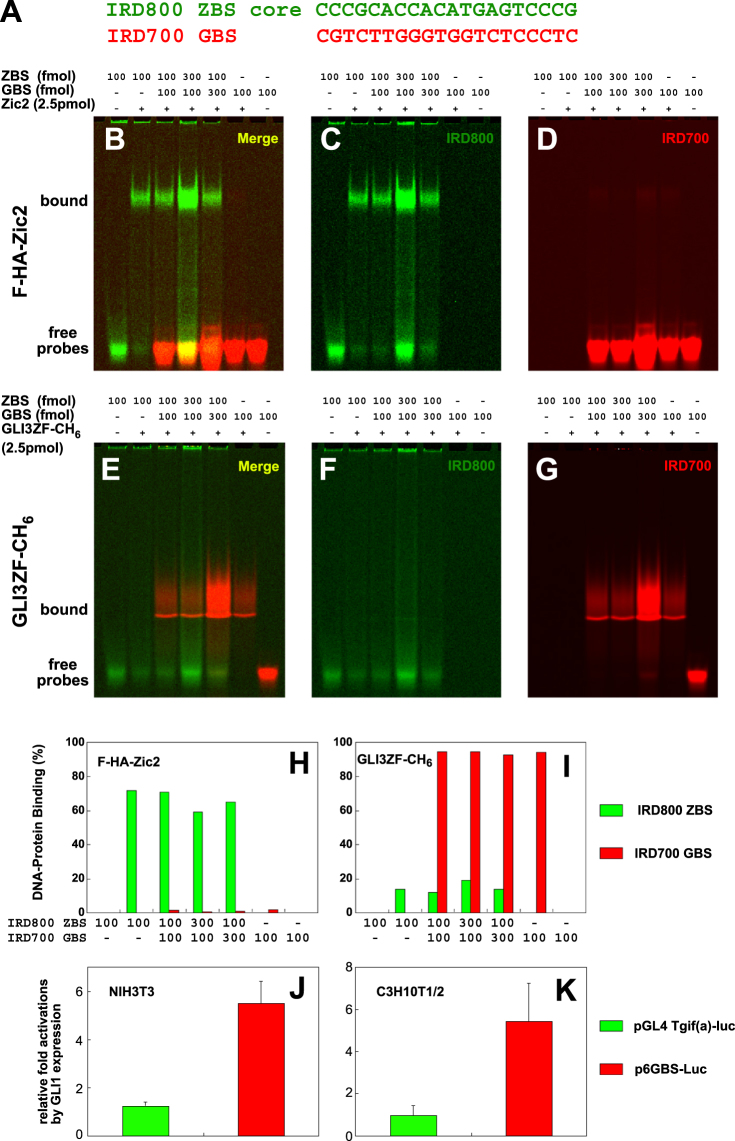
Differences in the DNA binding properties between the Zic2 and GLI1 ZFDs. (**A**) Sequence of two duplex DNA probes used for this experiment. Two different probes, IRD800 ZBS (Green) and IRD700 GBS (Red), were incubated with *Zic2* (**B**,**C**,**D** and **H**) protein or GLI3 zinc finger protein (**E**,**F**,**G** and **I**) alone (100 fmol) or together (100 fmol versus 100 fmol, or 100 fmol versus 300 fmol). Formed protein–DNA complexes were separated by native gel electrophoresis and analysed on the Odyssey Imaging Analyzer (Li-COR Biosciences) at 800 nm or 700 nm separately (**C**,**D**,**F** and **G**) and together (**B** and **E**). (**H** and **I**) Percentages of bound probes for F-HA-Zic2 (H) or GLI3ZF-CH_6_ (**I**) based on the densitometric quantification of images in (**C**,**D**,**F** and **G**). (**J** and **K**) GLI1 transcriptional activation was assayed using the pGL4 Tgif(a)-Luc and GBS-containing  luciferase reporter plasmid (p6GBS-Luc) in NIH3T3 and C3H10T1/2 cell lines. The averages and standard errors for three independent experiments of three samples are shown.

We previously showed that the mouse Zic2 zinc finger domain (ZFD) binds to a GLI binding sequence (GBS, 5′-CGTCTTGGGTGGTCTCCCTC-3′)^31,32^ with a lower affinity than that of the GLI3 ZFD^18^. To compare the relative binding affinity of Zic2 protein and GLI ZFD to GBS and ZBS core, we examined the target binding specificity by an EMS assay with a mixture of differentially end-labelled ZBS core and GBS (Fig. 3). Two differently modified duplex DNA probes IRD800-ZBS core and/or IRD700-GBS were mixed with F-HA-Zic2 protein or GLI3-ZF-CH_6_ proteins (Fig. 3A), and then the protein-DNA complexes were analysed by performing an EMS assay (Fig. 3B–G). It was observed that Zic2 was bound to more than 60% of the ZBS core probe but not to the GBS probe even in the presence of GBS at a three-fold excess (Fig. 3H). Conversely, GLI3-ZFD was preferentially bound to GBS (Fig. 3I). Accordingly, GLI1 did not induce the expression of the reporter gene in pGL4 Tgif(a)-Luc (Fig. 3J,K), but could induce the expression of a GBS-driven reporter gene in p6GBS-Luc.

### *Tgif1* expression is reduced in Zic2 knockdown mouse embryos

**Figure 4:**
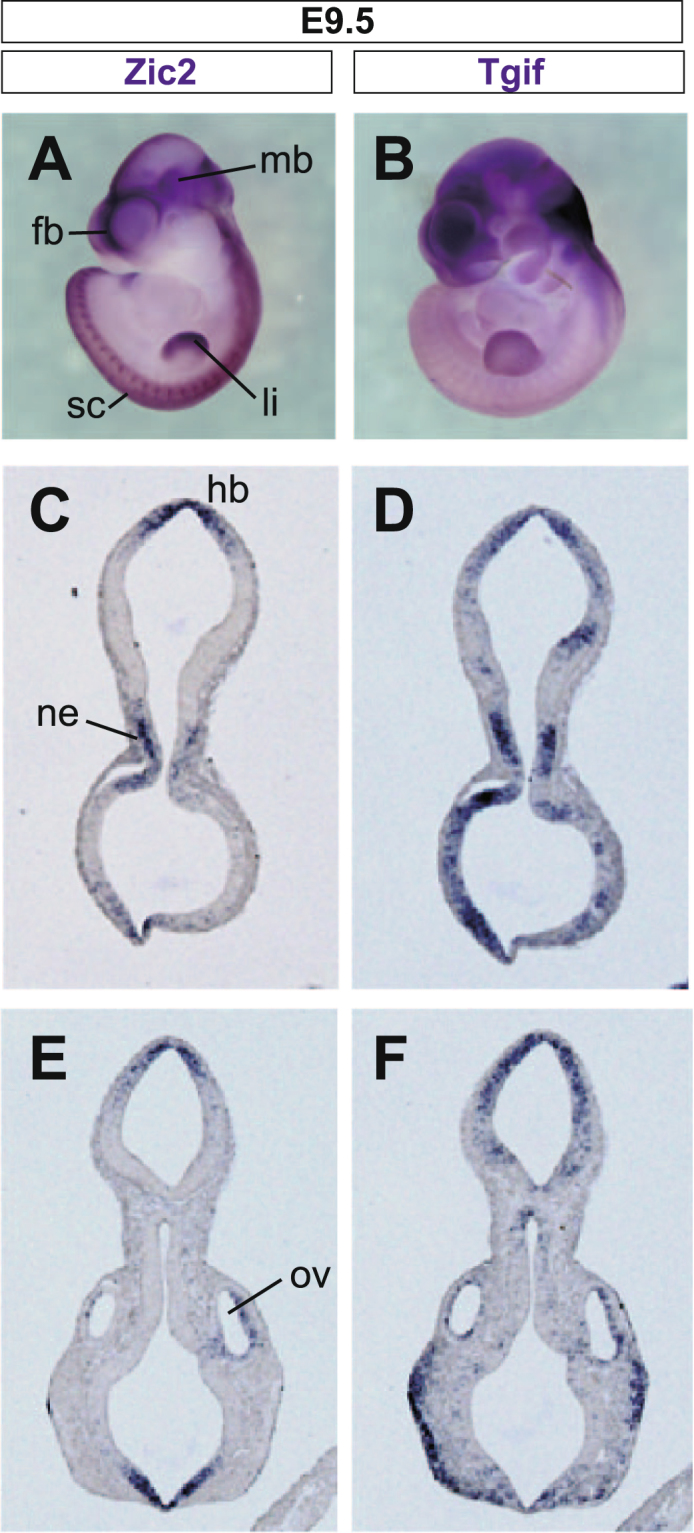
*Tgif1* gene expression during mouse development. Whole mount *in situ* hybridization was performed on E9.5 (**A** and **B**) mouse embryos. Both *Zic2* (**A** and **C**) and *Tgif1* (**B** and **D**) mRNA were expressed in the spinal cord (sc), forebrain (fb), midbrain (mb), and limb (li). Expression of *Zic2* (**C** and **E**) and *Tgif1* (**D** and **F**) in coronal sections of E9.5 mouse embryos. The expression of *Zic2* and *Tgif1* were detected in the roof of hindbrain (hb), the neuroepithelium (ne) of diencephalon, and wall of the optic vesicle (ov).

**Figure 5:**
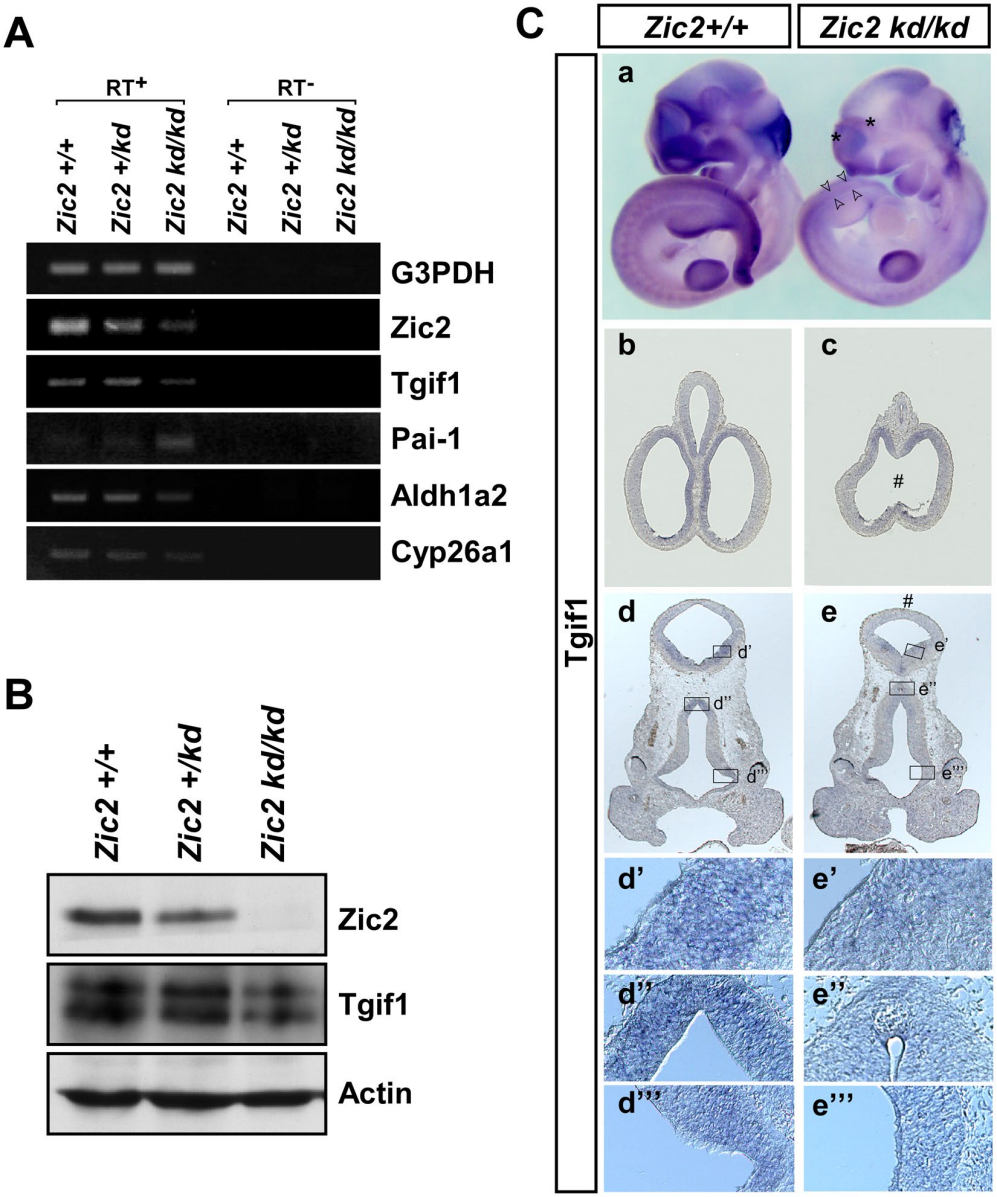
*Tgif1* expression in *Zic2* knockdown mice. (**A**) Total RNA was prepared from the head of E10.5 *Zic2*^+/+^*, Zic2*^+*/kd*^, and *Zic2*^*kd/kd*^ mouse embryos and subjected to RT-PCR analysis in the presence or absence of reverse transcriptase (RT^+^ or RT^−^, respectively). Specific primers were used for the detection of the indicated genes, and *Zic2, Tgif1*, *Pai-1* (a direct target of Tgif1), *Aldh1A2* (a downstream factor of Tgif1), *Cyp26A1* (a downstream factor of Tgif1), and *G3PDH* (a housekeeping gene control). (**B**) Total protein extracts from E14.5 *Zic2*^+/+^, *Zic2*^+*/kd*^, and *Zic2*^*kd/kd*^ mouse head for western blotting analysis using the indicated antibodies. Tgif1 protein levels normalized to those of actin were 100% in *Zic2*^+/+^, 74% in *Zic2*^+*/kd*^, and 61% in *Zic2*^*kd/kd*^ in densitometric measurement of the blots. (**C**) *In situ* hybridization of E10.5 (a), whole-mount) and E11.5 (b–e), sections) *Zic2*^+/+^ (left embryo in (a,b,d) and *Zic2*^*kd/kd*^ (right embryo in (a,c,e) mouse embryos. An antisense probe for *Tgif1* was used in the experiment. Tgif1 expression was reduced in the diencephalon (asterisk in a,d’,d”) where the two genes are expressed in an overlapping manner at E9.5 (Fig. 4). The telencephalic hindbrain roof plate showed abnormal shape (#). Spina bifida-like anomaly existed in the caudal region (arrowheads), where also the *Tgif1* expression was reduced.

In the developing mouse embryos, *Zic2* and *Tgif1* are expressed in the forebrain^33,34^. To compare their expression profiles, we carried out *in situ* hybridization analysis using E9.5 embryos (Fig. 4). The expression patterns of *Zic2* and *Tgif1* overlapped in the neuroepithelia of the dorsal telencephalon-, diencephalon-, and metencephalon-forming regions as well as in the optic vesicles. We then examined the expression of *Tgif1* in the *Zic2*-knockdown mutant mouse^26^. *Zic2* mRNA level was reduced in the homozygote embryo (*Zic2*^*kd/kd*^)^26^ in comparison to that of the wild type (*Zic2*^+/+^) at E10.5 (Fig. 5A). Tgif1 protein level was also reduced in the head of *Zic2*^*kd/kd*^ embryos at E14.5 (Fig. 5B). When we examined the *Tgif1* expression spatially in the E10.5 and E11.5 *Zic2*^*kd/kd*^ embryos by *in situ* hybridization, it became clear that *Tgif1* mRNA was reduced in the telencephalic and diencephalic regions where *Zic2* and *Tgif1* expression overlaps at E9.5 (Fig. 5C). These results indicate that Zic2 enhances *Tgif1* expression in the head of developing mice, suggesting that *Tgif1* is being regulated by Zic2.

Tgif1 is known to directly suppress *Pai-1* expression in mammalian cultured cells^7^ and to upregulate *Cyp26a1* and *Aldh1a2* in the development of zebrafish embryos^13^. As expected, *Pai-1* mRNA was increased whereas *Cyp26a1* and *Aldh1a2* mRNAs were decreased in the E10.5 *Zic2*^*kd/kd*^ embryo (Fig. 5A). These results suggested that Zic2 and Tgif1 are located in a common development-regulatory cascade, supporting the biological significance of Zic2-mediated regulation of *Tgif1* expression.

## Discussion

Zic2-binding sequences have been identified in promoter (near transcriptional start sites)^35–39^ and enhancer^19,20^ regions by yeast one-hybrid assays and ChIP-seq, or by systematic evolution of ligands by exponential enrichment (SELEX)^18,40^ and protein-binding microarrays^41^. The Zic2-binding motifs defined by these studies were contained in the ZIC2-binding site-containing region of the *Tgif1* promoters both in mice and humans (Supplementary Fig. S1), and the most proximal binding site (−1700 to −1670) matched with the ZIC3-binding site^40^ in a computer-assisted search. However, no matches were found for the other Zic2 binding sequences (−1728 to −1709 and −1830 to −1800) or corresponding human sequences. Because there were clear ChIP-seq peaks in mouse and human experiments, the current matrix may not be sufficient for the precise prediction of ZBS.

**Figure 6:**
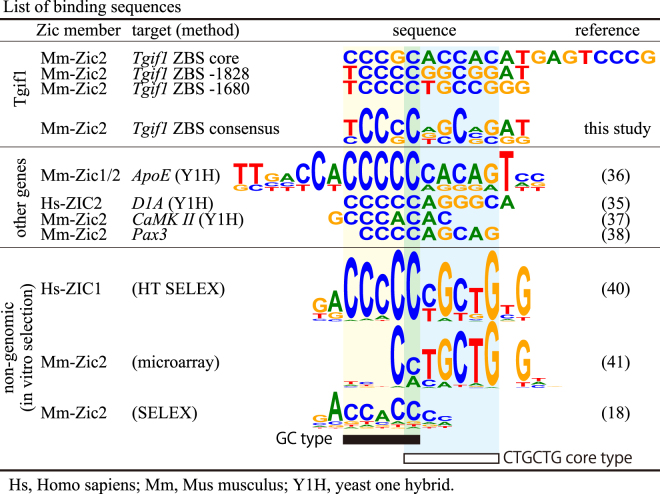
List of Zic2-binding sequences. Zic2-binding sequences in Tgif1 (top), other genes (middle), and non-genomic ones (bottom) are listed. *Hs*, human; *Mm*, mouse; *Y1H*, yeast one-hybrid assay. The frequencies appeared in the target sequences are indicated by WebLogo^58^.

In view of the currently available Zic2-binding sequences (Fig. 6), the binding sequence identified near the transcriptional start site always contain a stretch of 3–6 G or C nucleotides. On the other hand, another type of binding consensus that contains a core sequence of CTGCTG have been reported using an oligonucleotide-fixed microarray analysis for mouse Zic2-ZFD^41^. This sequence was similar to that obtained by a ChIP-seq analysis for mouse Zic3, raising the possibility that ZIC family proteins can target two types of sequences. In this regard, it is interesting that the ZIC1 and ZIC3 consensus sequences defined by a high-throughput SELEX analysis contains both a stretch of C nucleotides and the CTGCTG core motif adjacently (Fig. 6)^40^. Although this type of analysis has not been carried out for the Zic2-ZFD, it is possible that the optimized Zic2 target sequence is similar to that of ZIC1 and ZIC3, considering the highly conserved ZFD sequences among Zic1, ZIC2, and ZIC3^42^. Further clarification of the Zic2 binding sequences would involve a comparative analysis of the known target sequences and a comprehensive analysis performed by the combined use of ChIP-seq and an *in vitro* binding assay.

We also showed that Zic2 binds more preferentially to the *Tgif1* ZBS than GBS. In terms of DNA binding affinity, previous studies have shown that mouse Zic2 -ZFD binds GBS with lower affinity than human GLI3 ZFD (*K*_*d*_: Zic2 4.8 × 10^−8^; GLI3, 8.5 × 10^−9^ M)^18^, and mouse Zic3 binds the 5′-CCCGCTGGG-3′ sequence with a high affinity (*Kd* = 2.4 × 10^−9^ M). Interestingly, Drosophila Opa (*Zic* homologue) ZFD also showed a lower affinity to GBS than Drosophila Ci (*Gli* homologue) ZFD, and both Opa and mouse Zic2ZFD showed more binding to a SELEX-determined Opa-binding sequence (5′-GACCCCCCCG-3′) than GBS^43^. However, Ci-ZFD still binds the Opa-binding sequence with an approximately 25-fold higher affinity than Opa itself^43^. By contrast, Zic2-ZFD and GLI1-ZFD showed distinct binding preferences to GBS and *Tgif1* ZBS in our comparative binding assay (Fig. 4). Taken together, these results indicate that Zic and GLI proteins can have their own optimized target sequence while also sharing a common target with lower affinity. To our knowledge, *Tgif1* ZBS are the first target sequences that have been experimentally proven to show higher affinity to Zic-ZFD than GLI-ZFD. Although many studies independently established that the Zic target sequences were different from the GBS, there have been no comprehensive studies to show the binding specificity and affinity differences among the GLI-GLIS-ZIC superfamily proteins^44,45^.

Because the *Tgif1* 5′ flanking region mediates a clear Zic2/ZBS-dependent transcriptional activation in cultured mammalian cells, this region can be used for the functional evaluation of ZIC2. Furthermore, the *Tgif1* 5′ flanking region was cloned into a luciferase construct to demonstrate the ZIC2 functional impairment caused by a mutation (ZIC2 R409P) identified in a patient with schizophrenia^46^.

By organizing the currently known data on Zic2 and Tgif1 in relation to forebrain development, we can identify notable contact points between the two genes. Firstly, the spatial expression profiles of Zic2 and Tgif1 highly overlap in the developing nervous system just after anterior neuropore closure in mouse at E9.5 stage. Additionally, both genes are highly expressed in the developing epiblast, mesoderm, and neural plate at stages E6–8.5^25,33,34^. Secondly, both genes are known to influence the Nodal/TGF-β and SHH signalling. In mouse development, the PrCP migrate out from the anterior primitive streak and locate beneath the anterior neural plate around E7.5–7.75. The PrCP cells are the essential organizing centre for midline specification of the brain and facial structures because HPE is caused in amphibians and chicks by the removal of PrCP cells from their embryos^47^. Nodal signalling is required for proper PrCP development^48^. PrCP cells secretes SHH that is essential for the development of the ventral forebrain and the maintenance of PrCP^49^. Zic2 and Tgif1 act downstream of Nodal signalling and can commonly interact with SMAD2^7,25^. Thus, it is likely that Zic2 and Tgif1 share a common role in the pathogenesis of HPE, at least in part. In addition to Nodal signalling, both Zic family proteins and Tgif1 influence the SHH signalling through the regulation of Gli family proteins, and RA signalling^10,13,15,18,24,28,50,51^, both of which are implicated in HPE^2^.

Lastly, this study provided evidence for the direct regulation of *Tgif1* expression by Zic2. This regulatory relationship in conjunction with current studies show critical linkages among the HPE-causative genes. Further clarification of the regulatory relationships among the HPE-causative genes and HPE-associated signalling components would provide us a better overall picture regarding the aetiology of HPE.

## Methods

### Animals

We used CD1 (ICR) mice obtained from Nihon SLC (Shizuoka, Japan) for the *in situ* hybridization assay. Zic2-knockdown mice were generated and maintained as described^26^. The mice were maintained by the Laboratory Animal Facility, Research Resource Center, RIKEN BSI. All animal experiments were approved by Animal Experiment Committees at the RIKEN Brain Science Institute and Animal Care and Use Committee of Nagasaki University, and carried out in accordance with the guidelines for animal experimentation in RIKEN and Nagasaki University.

### Cell Lines

A40 cells were maintained in a 1:1 mixture of Dulbecco’s modified Eagle’s medium (DMEM) and Ham’s F12 without phenol red, supplemented with 10% foetal bovine serum (FBS). For protein production, expression vectors were transfected with Lipofectamine with PLUS reagent (Invitrogen). 293T, NIH3T3, and C3H10T1/2 cell lines were maintained in DMEM supplemented with 10% FBS. For the luciferase reporter assay, the transfection was performed with Lipofectamine with PLUS reagent (Invitrogen) as previously described^22^.

### Plasmids

The mammalian expression vectors pEF-RL, pEF-Zic2, and the N-terminal double-tagged expression plasmid, pCMV-F-HA-Zic2, were previously described^22,23^. The *E. coli* expression vector pET-GLI3 ZF was previously described^18^. The luciferase reporter plasmids pGL4 Tgif(a) and pGL4 Tgif(b) were constructed by inserting PCR-cloned mouse genomic DNA containing the *Tgif1* 5′ flanking region with the following primers: −1872 to +47, 5′-AAA GAT ATC CTC GGT CCA GCT CCT CCT CCC CG-3′ and 5′-AAA AGA TCT CTC AGC TCC TTT GTT TCC CGC TCC-3′; −1648 to +47, 5′-AAA GAT ATC GGT TTG CAG AGG GGG CTT GCC AG-3′ and 5′-AAA AGA TCT CTC AGC TCC TTT GTT TCC CGC TCC-3′. The EcoRV and BglII sites of pGL4 (Promega) were used for the cloning. For the *Tgif1 in situ* hybridization probe, mouse cDNA (RACE-Ready Mouse cDNA, Clontech) was amplified by performing PCR using the following primers: 5′-CTG ATG CTG CAA CAA GAC CCT TCT-3′ and 5′-TAA GCT GTG AGT TTG GCC TGA AGC-3′. The expected PCR product of 1000 bp was cloned into the pGEM-T vector (Promega).

### ChIP cloning and ChIP-PCR

We used a modified strategy of ChIP cloning that was previously^52^. A40 cells (four 10 cm dishes for a reaction) were transfected with pCMV-F-HA-Zic2 or a control empty plasmid pCMV-F-HA. For the DNA-Zic2 crosslink, formaldehyde was added to the cells in the growth medium to a final concentration of 1% 24 h post-transfection, and incubated for 10 min at room temperature, the crosslinking was quenched by adding 1/10 volume of 1.25 M Glycine. Cells were washed with PBS, and the harvested cells were resuspended in 0.5 ml lysis buffer (50 mM Tris-HCl pH 8.0, 300 mM NaCl, 10% glycerol, 0.5% NP-40 [Nonidet-P40], 1 mM DTT, 0.1 mM EDTA, and 1 mM PMSF). The cells were then sonicated 5 times for 30 sec each at the maximum setting (COSMO BIO) and centrifuged for 15 min at 14,000 × g. The Zic supernatant was mixed with anti-HA antibody-conjugated agarose beads (20 μl) for 6 h at 4 °C. The beads were washed with lysis buffer containing 500 mM NaCl, and the bound DNA-Zic2 complexes were eluted by the addition of HA peptide (100 μg/ml). The elution was mixed with anti-FLAG antibody-conjugated agarose beads (10 μl) for 6 h at 4 °C. The beads were washed with lysis buffer containing 500 mM NaCl, and the DNA-Zic2 complexes were decrosslinked in decrosslinking buffer (50 mM Tris-HCl pH 8.0, 1% SDS, 10 mM EDTA) for 6 h at 65 °C. The reactions containing the released DNA fragments were removed to a new tube, and the contaminating proteins were removed with two volumes of phenol/chloroform/isoamyl alcohol extraction. The DNA fragments were concentrated via ethanol precipitation, and were then blunted with mungbean nuclease. After a phenol/chloroform/isoamyl alcohol extraction, DNA fragments were concentrated via ethanol precipitation and cloned into the EcoRV site of the pBluescript SK(+) plasmid. The nucleotide sequencing was performed by the Research Resource Center at RIKEN BSI.

The isolated sequences were validated by ChIP-PCR because the ChIP system often includes background noises, which are generated during the crosslinking of nonspecific DNA–protein complexes and immuno-affinity isolation of DNA–protein complexes. For ChIP-PCR, A40 cells (10 cm dish per reaction) were treated with formaldehyde and the crosslinking was quenched by the addition of 1/10 volume of 1.25 M Glycine, and cell extract was prepared in the same manner as for ChIP cloning. Anti-Zic2 antiserum^53^ or preimmune serum (2 μl) were added to the cell extracts together with protein A sepharose beads (10 μl) for 6 h at 4 °C. The beads were washed with lysis buffer containing 500 mM NaCl, and the DNA-Zic2 complexes were decrosslinked in decrosslinking buffer for 6 h at 65 °C. The DNA mixtures were prepared by phenol/chloroform/isoamyl alcohol extraction and ethanol precipitation. PCR was performed using the DNA mixtures and *Tgif1* 5′ flanking region-specific primers (5′-CCA GGG AGA ACC CAA CGG CTG GC-3′ and 5′-TCA CCG CCG GGT CCG GAC CCG GC-3′). The anti-Zic2 antiserum also recognizes Zic1^53^. However, the initial ChIP-sequencing using F-HA-Zic2 warrants the Zic2-specificity.

The sequences from 302 clones were analysed with the BLAST search against the mouse genome database. The sequences from 26 clones were proximally located within 20,000 bp from +1 of known genes. The other sequences were mapped to intergenic regions or near uncharacterized genes (data not shown).

The plasmid harbouring the Tgif1 5′ Zic2 binding regions, pBS-Tgif1, containing the region –1866 to −1649 (from the transcriptional start site of *Tgif1*, +1) in the forward direction from T3 promoter of the pBluescript SK(+) plasmid was also generated.

### DNase I DNA footprinting

DNA footprinting was performed using purified F-HA-Zic2 proteins^22^ and DNA fragments with the *Tgif1* 5′ region, which was digested from the pBS-Tgif plasmid. The fragment was digested at the BamHI and HindIII sites, and was labelled with ^32^P with T4 polynucleotide kinase. After the reaction, the BamHI side was digested at the EcoRI inner site and separated by native gel electrophoresis^54^. The ^32^P labelled 1 nM DNA fragments were incubated in the presence or absence of 12.5 nmol F-HA-Zic2 protein in the reaction buffer (20 mM Tris-HCl pH8.0, 3 mM MgCl_2_, 5 mM CaCl_2_, 100 mM NaCl, 100 mM DTT, 0.1 mM EDTA, and 50 μg/ml bovine serum albumin) for 30 min at 25 °C. DNase I (20 ng) was added and incubate for 60 s at 25 °C. The samples were extracted with phenol/chloroform/isoamyl alcohol, and precipitated with ethanol. DNA was analysed by 8 M urea 8% polyacrylamide gel electrophoresis together with marker DNA, which was digested at GTP by the Maxam-Gilbert method^54^. The gel was analysed with the Bioimaging Analyzer BAS2500 (Fuji).

### Gel shift assay

The gel shift assay was performed as described previously^22^. The duplex DNA probes were amplified by PCR or annealed using the following synthesized IRD-modified oligonucleotides: probe for the −1866 to −1649 region, 5′-IRD700-CCA GCT CCT CCT CCC CGA GTC TG-3′ and 5′-IRD700-GCC CTG GCG CAG CGC TTT CCG GG-3′; probe for the −1709 to −1649 region, 5′-CAC CGG GGC GCA GCA GCT GA-3′ and 5′-IRD700-GCC CTG GCG CAG CGC TTT CCG GG-3′; probe for the −1866 to −1760 region, 5′-IRD700-CCA GCT CCT CCT CCC CGA GTC TG-3′ and 5′-GCT CTG GGT TTT TGG TGT TT-3′; probe for the −1728 to −1709 region (mouse ZBS), 5′-IRD700-CCC GCA CCA CAT GAG TCC CG-3′ and 5′-IRD700-CGG GAC TCA TGT GGT GCG CC-3′, or 5′-IRD800-CCC GCA CCA CAT GAG TCC CG-3′ and 5′-IRD800-CGG GAC TCA TGT GGT GCG CC-3′; probe for the GBS, 5′-IRD800-CGT CTT GGG TGG TCT CCC TC-3′ and 5′-IRD800-GAG GGA GAC CAC CCA AGA CG-3′. Proteins used for this assay were described previously^18,22^.

### Luciferase Reporter Assay

Luciferase reporter assays were performed as previously described^18^. The luciferase reporter plasmid pGL4 Tgif(a) and pGL4 Tgif(b) (100 ng) were co-transfected with pEFBOS or pEFBOS-Zic2 (100 ng), and pEF-RL (5 ng). Luciferase activity was measured according to the manufacturer’s recommendations (Promega) using a luminometer, the Minilumat LB 9506 (Berthold).

### Bioinformatics analysis

ChIP-seq peaks were displayed using the Cistrome database (http://cistrome.org/db/#/)^55^, ENCODE database (https://www.encodeproject.org/)^56^, and the UCSC browser (https://genome.ucsc.edu/). A computer-assisted Zic family binding sites search was carried out using the MatInspector program (Genomatix, Munich, Germany). A homology search was carried out with the NCBI BLAST (https://blast.ncbi.nlm.nih.gov/Blast.cgi).

### *In situ* hybridization and RT-PCR analyses

Whole-mount and section *in situ* hybridizations were performed as described^33^. RT-PCR analysis was performed as previously described^57^ using the following primers: *Zic2* (5′-TCT CTGGAGCAC GTC GGC GG-3′, 5′-CTT GCT ATG CTC GCT TCC CGG AC-3′), *Tgif1* (5′-GAG ACC AGT GTC TCC CAA ACC TCC-3′, 5′-AGA AGC TGG AAT CCA CTA AAA TCC-3′), *G3PDH* (5′-CCG GTG CTG AGT ATG TCG TGG AGT CTA C-3′, 5′-CTT TCC AGA GGG GCC ATC CAC AGT CTT C-3′), *Pai-1* (5′-TAC CCC TCC GAG AAT CCC ACA CAG-3′, 5′-GCT GGA CAA AGA TGG CAT CCG CAG-3′), *Aldh1A2* (5′-CCA TTG GAG TGT GTG GAC AG-3′, 5′-GTC CAA GTC AGC ATC TGC AA-3′) and *Cyp26A1* (5′-TTC GGG TTG CTC TGA AGA CT-3′, 5′-GGG AGA TTG TCC ACA GGG TA-3′).

### Western blotting

Western blotting with anti-Zic2 antibody was performed as previously described^22,53^. Anti-Tgif1 antibody was developed in the rat using a GST-fusion Tgif1 (340–690) protein. GST-Tgif1 was produced in *E. coli* using pGEX-Tgif1 (340–690) plasmid, and was purified as previously described^22^.

### Data availability

All data generated or analysed during this study are included in this published article (and its Supplementary Information files).

## Electronic supplementary material

Supplementary information

## References

[1] Geng X, Oliver G (2009). Pathogenesis of holoprosencephaly. J Clin Invest.

[2] Petryk A, Graf D, Marcucio R (2015). Holoprosencephaly: signaling interactions between the brain and the face, the environment and the genes, and the phenotypic variability in animal models and humans. Wiley Interdiscip Rev Dev Biol.

[3] Roessler E, Muenke M (2010). The molecular genetics of holoprosencephaly. Am J Med Genet.

[4] Paulussen AD (2010). The unfolding clinical spectrum of holoprosencephaly due to mutations in SHH, ZIC2, SIX3 and TGIF genes. Eur J Hum Genet.

[5] Roessler E, Velez JI, Zhou N, Muenke M (2012). Utilizing prospective sequence analysis of SHH, ZIC2, SIX3 and TGIF in holoprosencephaly probands to describe the parameters limiting the observed frequency of mutant genexgene interactions. Mol Genet Metab.

[6] Gripp KW (2000). Mutations in TGIF cause holoprosencephaly and link NODAL signalling to human neural axis determination. Nat Genet.

[7] Wotton D, Lo RS, Lee S, Massague J (1999). A Smad transcriptional corepressor. Cell.

[8] Seo SR (2004). The novel E3 ubiquitin ligase Tiul1 associates with TGIF to target Smad2 for degradation. The EMBO journal.

[9] Bertolino E, Reimund B, Wildt-Perinic D, Clerc RG (1995). A novel homeobox protein which recognizes a TGT core and functionally interferes with a retinoid-responsive motif. J Biol Chem.

[10] Bartholin L (2006). TGIF inhibits retinoid signaling. Mol. Cell Biol.

[11] Castillo, H. A. *et al*. Insights into the organization of dorsal spinal cord pathways from an evolutionarily conserved raldh2 intronic enhancer. *Development* (*Cambridge, England*) **137**, 507–518. 10.1242/dev.043257 PMC4074295 20081195

[12] Powers SE (2010). Tgif1 and Tgif2 regulate Nodal signaling and are required for gastrulation. Development (Cambridge, England).

[13] Gongal PA, Waskiewicz AJ (2008). Zebrafish model of holoprosencephaly demonstrates a key role for TGIF in regulating retinoic acid metabolism. Hum Mol Genet.

[14] Taniguchi K, Anderson AE, Sutherland AE, Wotton D (2012). Loss of Tgif function causes holoprosencephaly by disrupting the SHH signaling pathway. PLoS Genet.

[15] Taniguchi K (2017). Genetic and Molecular Analyses indicate independent effects of TGIFs on Nodal and Gli3 in neural tube patterning. Eur J Hum Genet.

[16] Aruga J (2004). The role of Zic genes in neural development. Mol Cell Neurosci.

[17] Houtmeyers R, Souopgui J, Tejpar S, Arkell R (2013). The ZIC gene family encodes multi-functional proteins essential for patterning and morphogenesis. Cell Mol Life Sci.

[18] Mizugishi K, Aruga J, Nakata K, Mikoshiba K (2001). Molecular properties of Zic proteins as transcriptional regulators and their relationship to GLI proteins. J Biol Chem.

[19] Frank CL (2015). Regulation of chromatin accessibility and Zic binding at enhancers in the developing cerebellum. Nat Neurosci.

[20] Luo Z (2015). Zic2 is an enhancer-binding factor required for embryonic stem cell specification. Mol Cell.

[21] Matsuda K (2017). ChIP-seq analysis of genomic binding regions of five major transcription factors highlights a central role for ZIC2 in the mouse epiblast stem cell gene regulatory network. Development.

[22] Ishiguro A, Ideta M, Mikoshiba K, Chen DJ, Aruga J (2007). ZIC2-dependent transcriptional regulation is mediated by DNA-dependent protein kinase, poly(ADP-ribose) polymerase, and RNA helicase A. J Biol Chem.

[23] Ishiguro A, Aruga J (2008). Functional role of Zic2 phosphorylation in transcriptional regulation. FEBS Lett.

[24] Koyabu Y, Nakata K, Mizugishi K, Aruga J, Mikoshiba K (2001). Physical and functional interactions between Zic and Gli proteins. J Biol Chem.

[25] Houtmeyers R (2016). Zic2 mutation causes holoprosencephaly via disruption of NODAL signalling. Hum Mol Genet.

[26] Nagai T (2000). Zic2 regulates the kinetics of neurulation. Proc Natl Acad Sci USA.

[27] Warr N (2008). Zic2-associated holoprosencephaly is caused by a transient defect in the organizer region during gastrulation. Hum Mol Genet.

[28] Maurus D, Harris WA (2009). Zic-associated holoprosencephaly: zebrafish Zic1 controls midline formation and forebrain patterning by regulating Nodal, Hedgehog, and retinoic acid signaling. Genes Dev.

[29] Tomooka Y, Aizawa S (1998). Cell lines established from fetal brains of p53-deficient mice. Cell Str Func.

[30] Najafabadi HS (2015). C2H2 zinc finger proteins greatly expand the human regulatory lexicon. Nat Biotechnol.

[31] Kinzler KW, Ruppert JM, Bigner SH, Vogelstein B (1988). The GLI gene is a member of the Kruppel family of zinc finger proteins. Nature.

[32] Kinzler KW, Vogelstein B (1990). The GLI gene encodes a nuclear protein which binds specific sequences in the human genome. Mol Cell Biol.

[33] Nagai T (1997). The expression of the mouse Zic1, Zic2, and Zic3 gene suggests an essential role for Zic genes in body pattern formation. Dev Biol.

[34] Jin JZ, Gu S, McKinney P, Ding J (2006). Expression and functional analysis of Tgif during mouse midline development. Dev Dyn.

[35] Yang Y, Hwang CK, Junn E, Lee G, Mouradian MM (2000). ZIC2 and Sp3 repress Sp1-induced activation of the human D1A dopamine receptor gene. J Biol Chem.

[36] Salero E, Perez-Sen R, Aruga J, Gimenez C, Zafra F (2001). Transcription factors Zic1 and Zic2 bind and transactivate the apolipoprotein E gene promoter. J Biol Chem.

[37] Sakurada T, Mima K, Kurisaki A, Sugino H, Yamauchi T (2005). Neuronal cell type-specific promoter of the alpha CaM kinase II gene is activated by Zic2, a Zic family zinc finger protein. Neurosci Res.

[38] Sanchez-Ferras O, Bernas G, Laberge-Perrault E, Pilon N (2014). Induction and dorsal restriction of Paired-box 3 (Pax3) gene expression in the caudal neuroectoderm is mediated by integration of multiple pathways on a short neural crest enhancer. Biochim Biophys Acta.

[39] Zhu P (2015). ZIC2-dependent OCT4 activation drives self-renewal of human liver cancer stem cells. J Clin Invest.

[40] Jolma A (2013). DNA-binding specificities of human transcription factors. Cell.

[41] Badis G (2009). Diversity and complexity in DNA recognition by transcription factors. Science.

[42] Aruga J (1996). The mouse zic gene family. Homologues of the Drosophila pair-rule gene odd-paired. J Biol Chem.

[43] Sen A, Stultz BG, Lee H, Hursh DA (2010). Odd paired transcriptional activation of decapentaplegic in the Drosophila eye/antennal disc is cell autonomous but indirect. Dev Biol.

[44] Hatayama M, Aruga J (2010). Characterization of the tandem CWCH2 sequence motif: a hallmark of inter-zinc finger interactions. BMC Evol Biol.

[45] Layden MJ, Meyer NP, Pang K, Seaver EC, Martindale MQ (2010). Expression and phylogenetic analysis of the zic gene family in the evolution and development of metazoans. Evodevo.

[46] Hatayama M (2011). Zic2 hypomorphic mutant mice as a schizophrenia model and ZIC2 mutations identified in schizophrenia patients. Sci Rep.

[47] Li H, Tierney C, Wen L, Wu JY, Rao Y (1997). A single morphogenetic field gives rise to two retina primordia under the influence of the prechordal plate. Development (Cambridge, England).

[48] Andersson O, Reissmann E, Jornvall H, Ibanez CF (2006). Synergistic interaction between Gdf1 and Nodal during anterior axis development. Dev Biol.

[49] Aoto K (2009). Mouse Shh is required for prechordal plate maintenance during brain and craniofacial morphogenesis. Dev Biol.

[50] Quinn ME, Haaning A, Ware SM (2012). Preaxial polydactyly caused by Gli3 haploinsufficiency is rescued by Zic3 loss of function in mice. Hum Mol Genet.

[51] Drummond DL (2013). The role of Zic transcription factors in regulating hindbrain retinoic acid signaling. BMC Dev Biol.

[52] Weinmann AS, Farnham PJ (2002). Identification of unknown target genes of human transcription factors using chromatin immunoprecipitation. Methods.

[53] Inoue T, Ota M, Mikoshiba K, Aruga J (2007). Zic2 and Zic3 synergistically control neurulation and segmentation of paraxial mesoderm in mouse embryo. Dev Biol.

[54] Tagami H, Aiba H (1995). Role of CRP in transcription activation at Escherichia coli lac promoter: CRP is dispensable after the formation of open complex. Nucleic Acids Res.

[55] Mei S (2017). Cistrome Data Browser: a data portal for ChIP-Seq and chromatin accessibility data in human and mouse. Nucleic Acids Res.

[56] Consortium EP (2012). An integrated encyclopedia of DNA elements in the human genome. Nature.

[57] Fujimi TJ, Mikoshiba K, Aruga J (2006). Xenopus Zic4: conservation and diversification of expression profiles and protein function among the Xenopus Zic family. Dev Dyn.

[58] Crooks GE, Hon G, Chandonia JM, Brenner SE (2004). WebLogo: a sequence logo generator. Genome Res.

