# Supplementary material for: Link between the causative genes of holoprosencephaly: Zic2 directly regulates Tgif1 expression
Source: Sci Rep. 2018 Feb 1;8:2140. doi: 10.1038/s41598-018-20242-2 (PMC5794963; doi:10.1038/s41598-018-20242-2)

Supplementary information for Akira Ishiguro, Minoru Hatayama, Maky Ideta-Otsuka, and Jun Aruga "Link between the causative genes of holoprosencephaly, Zic2 directly regulates Tgif1 expression"

Supplementary Table S1

Results of ChIP cloning

Zic2 target		Position from +1*
Tgif1	*TG-interacting factor*	-1866 ~ -1649
Ssfa2	*Sperm specific antigen, Cs-1*	-305 ~ -179, -306 ~ -193
Pcolce2	*Procollagen C-endopeptidase enhancer 2*	-1 ~ +44
Ube2r2	*Ubiquitin-conjugating enzyme E2R2*	-381 ~ -71
Tef-1	*TEA domain family member-1, TEAD1*	-69 ~ +407
1810041L15Rik		-3495 ~ -3374
Ctsz	*Cathepsin Z*	-272 ~ -45
Ino80	*Homologue of yeast INO80*	-874 ~ -454
Mok	*MOK Protein Kinase (Renal tumour antigen, Rage)*	-3901 ~ -3847
Tnrc18	*Zinc finger protein 469*	-6612 ~ -5421
Chd1	*Chromodomain helicase DNA binding protein 1*	-991 ~ -795
Adgrg1	*G protein-coupled receptor 56 (Gpr56)*	-641 ~ -302
Med4	*Mediator Complex Subunit 4*	-1979 ~ -1658

*Positions from transcriptional start sites (+1).

Supplementary Figure legends

Fig. S1. ChIP-seq peaks around mouse and human *TGIF1* gene.

Mouse Zic2 ChIP-seq results using mouse mature (P60) (top) and immature (P7) (second line) cerebellar granule neurons[19] or mouse ES cells (ESC) (third line)[20]. GFP-human ZIC2-ZFD ChIP-seq results using HEK293 cells (HEK) (bottom)[30]. The thick black horizontal line on the yellow box indicates the location of the ChIP fragment obtained in this study or the syntenic region in the human chromosome. The thick red horizontal line on the red box indicates the location of the *Tgif1* Zic2 binding domain (-1866 to -1499) or the syntenic region in human chromosome. The vertical allow and arrowheads indicates the major ChIP peaks in a previous study in mouse and human, respectively. The horizontal arrows indicates the direction of transcription.

Fig. S2. Additional Zic2 binding sites.

(A) To confirm the two weak binding sites within the region -1866 to -1649, two probes for the regions -1709 to -1646 (in red) and -1866 to -1760 (in green) were used for the EMS assay. (B) Four unlabelled competitors (cp6–9) were used to evaluate for interferences. Only cp8 for the region -1688 to -1669 inhibited the formation of Zic2-DNA. (C) Six competitors (cp10–15) were assayed. Competitor cp11 for the region -1830 to -1811 inhibited the formation of the complex.

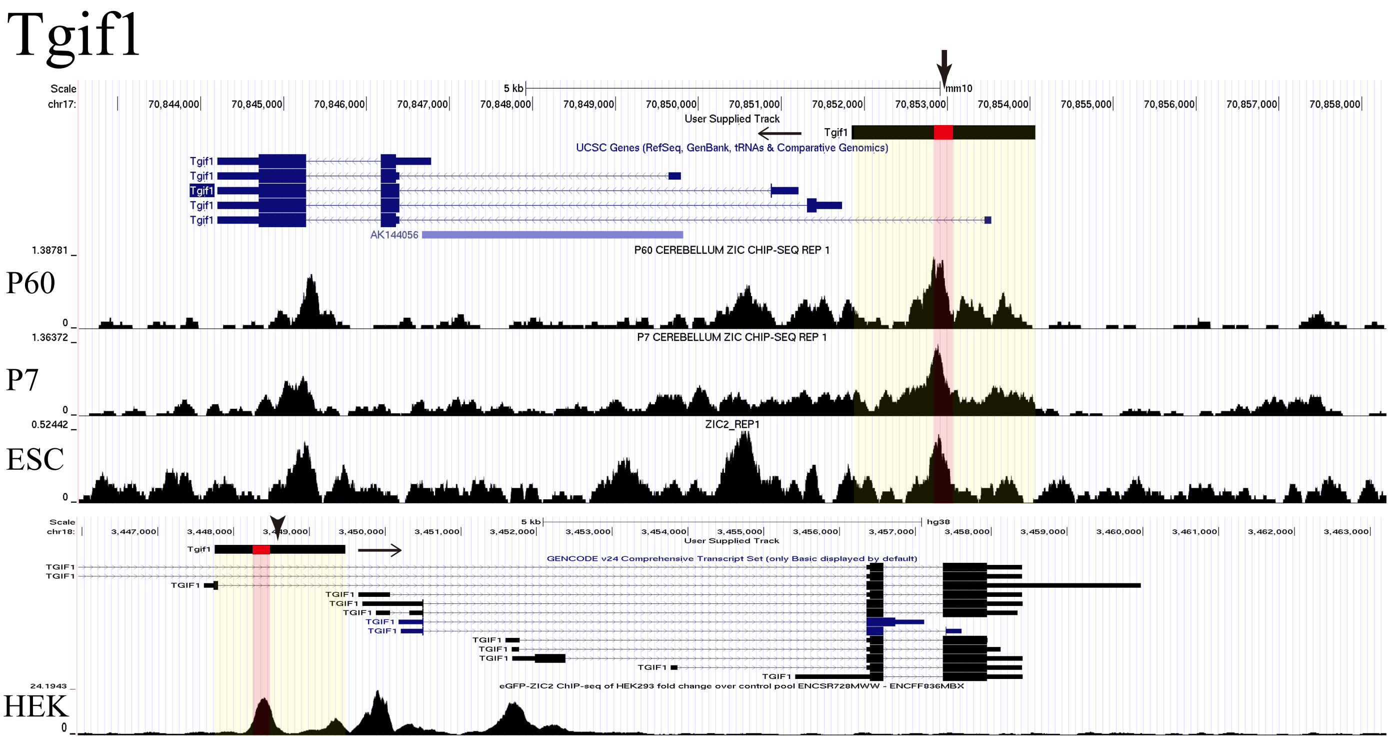
Tgif1
P60
P7
ESC
HEK
User Supplied Track
UCSC Genes (RefSeq, GenBank, tRNAs & Comparative Genomics)
P60 CEREBELLUM ZIC CHIP-SEQ REP 1
P7 CEREBELLUM ZIC CHIP-SEQ REP 1
ZIC2_REP1
GENCODE v24 Comprehensive Transcript Set (only Basic displayed by default)
eGFP-ZIC2 ChIP-seq of HEK293 fold change over control pool ENCSR728MWW - ENCFF836MBX

Supplementary Figure S1-2

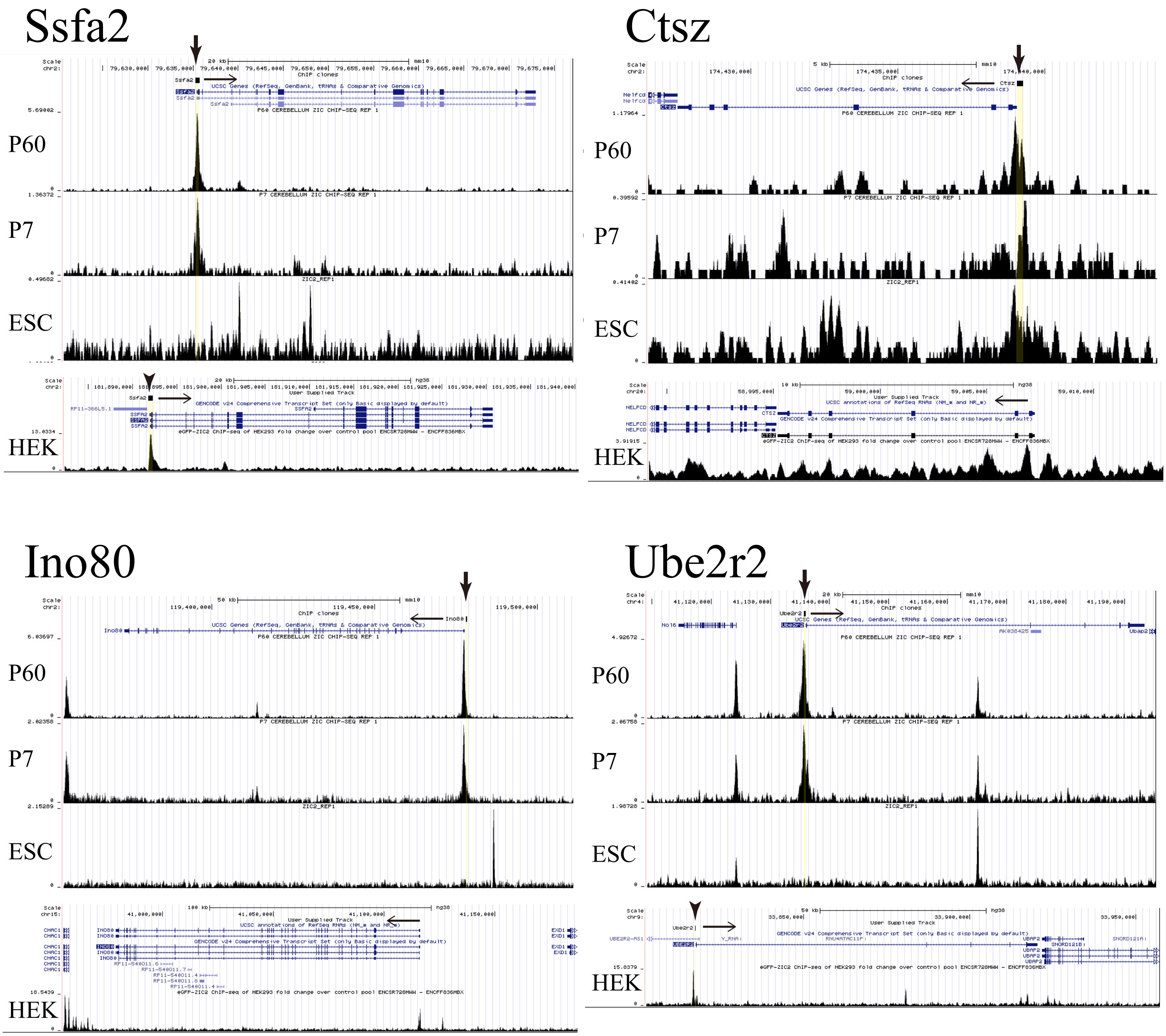

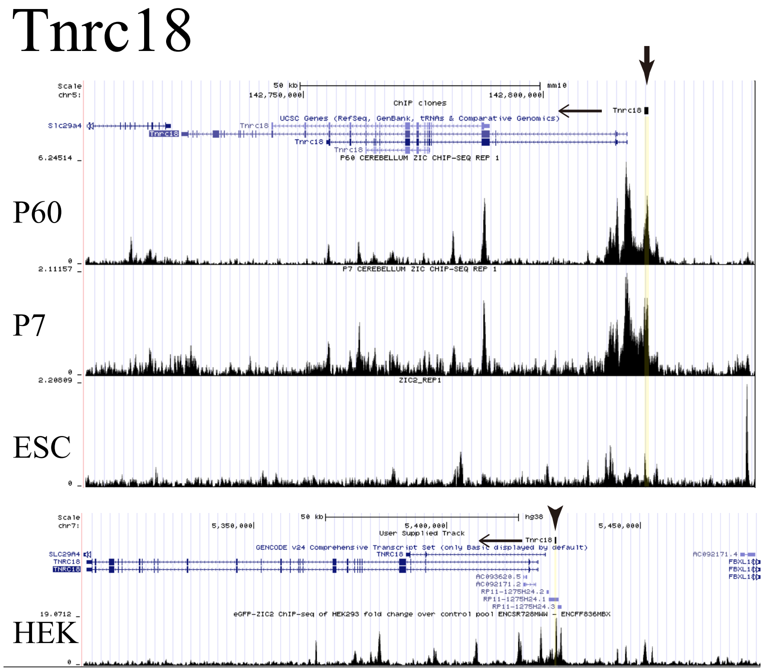
Tnrc18
P60
P7
ESC
HEK

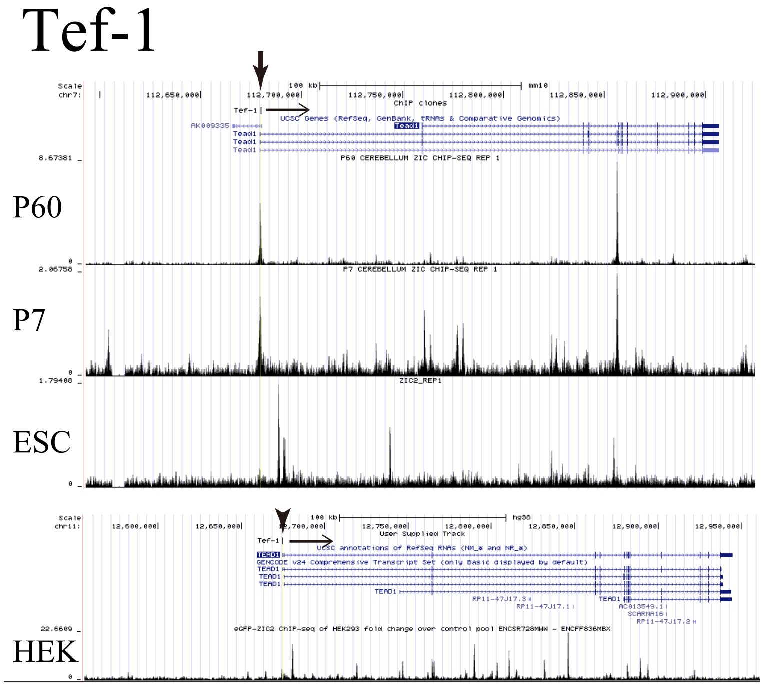
Tef-1
P60
P7
ESC
HEK

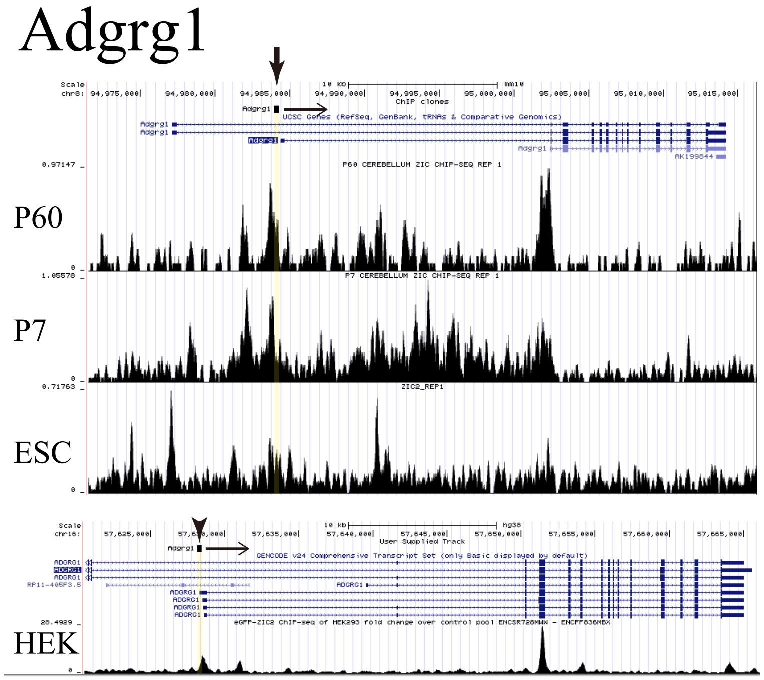
Adgrg1
P60
P7
ESC
HEK

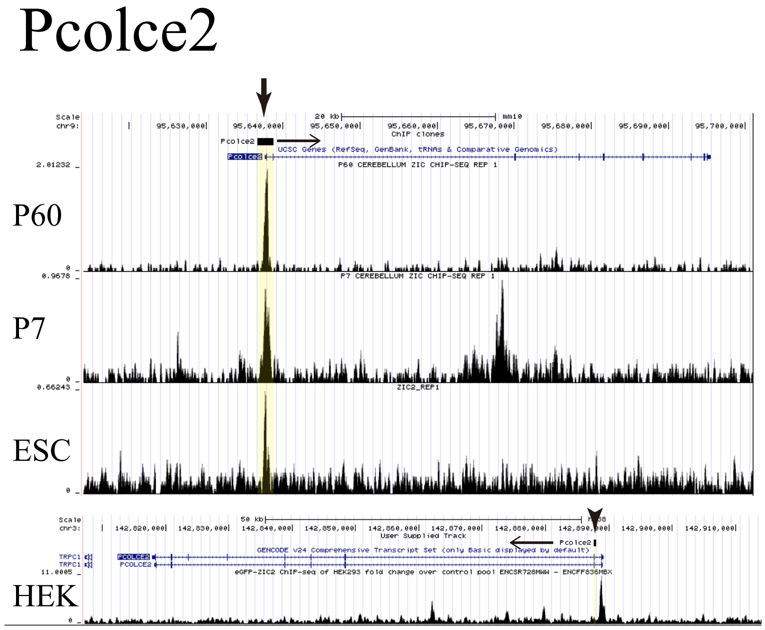
Pcolce2
P60
P7
ESC
HEK

Supplementary Figure S1-4

Mok

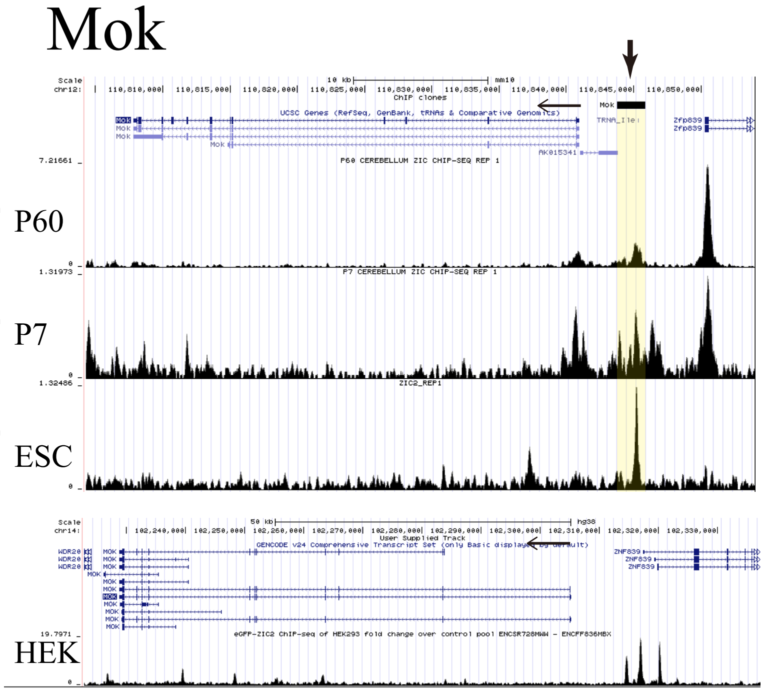

Med4

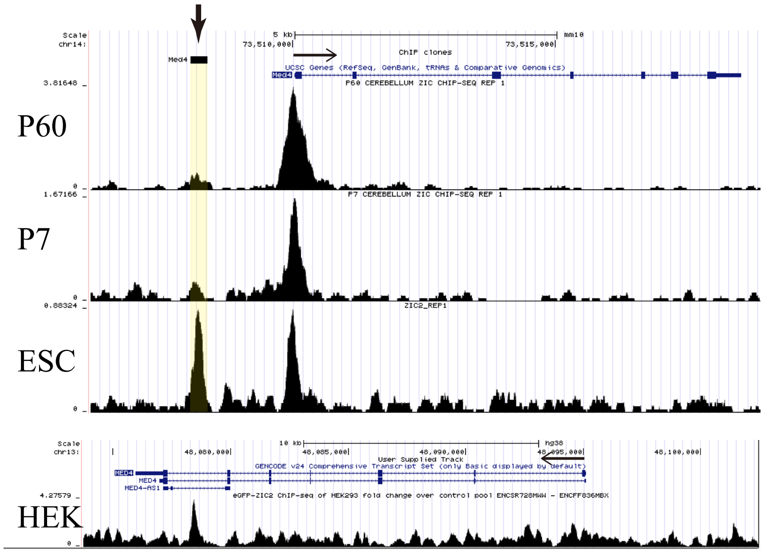

1810041L15Rik

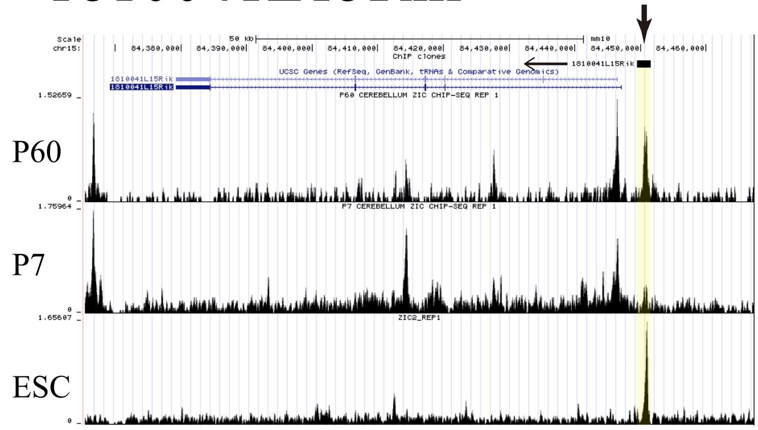

Chd1

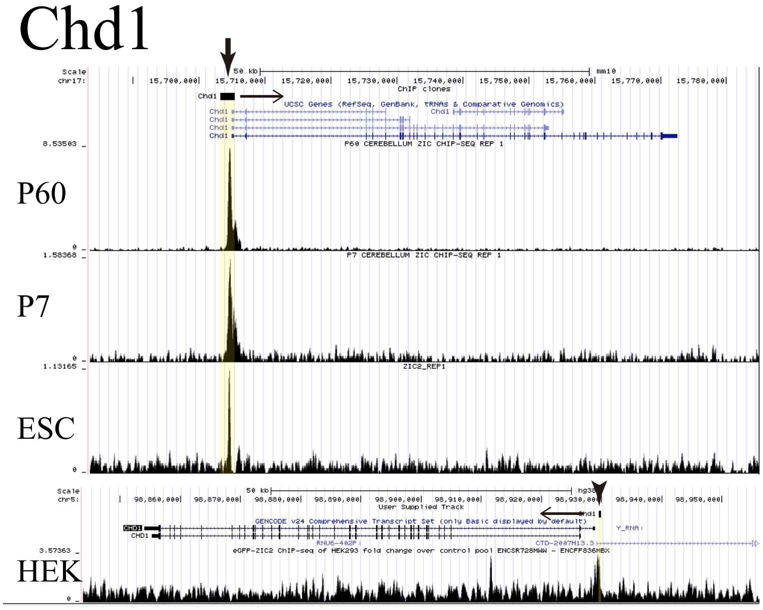

Supplementary Figure S2

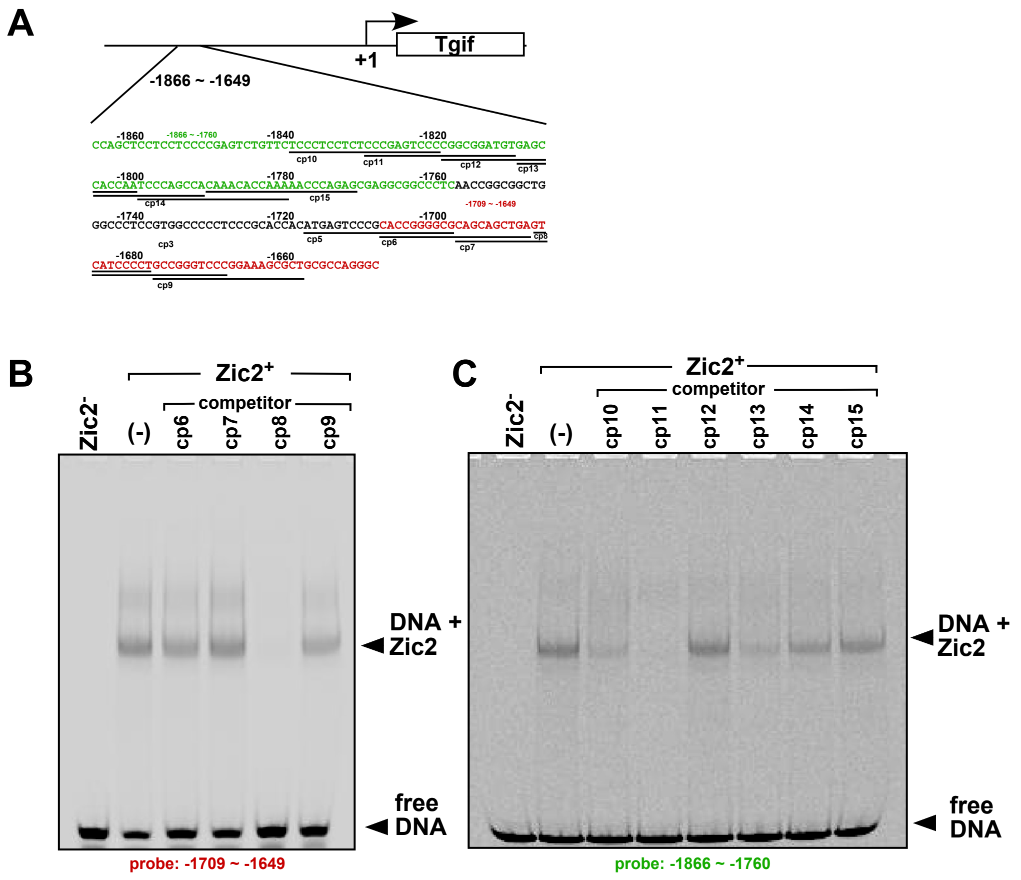

Supplement: Supplementary file 1 — Supplementary information [file 41598_2018_20242_MOESM1_ESM.pdf]
